# Community Women's Health Hub models in England: a mixed methods evaluation

**DOI:** 10.1186/s12875-025-03037-z

**Published:** 2025-12-16

**Authors:** K. Daniel, J. Bousfield, L. Hocking, L. Jackson, B. Taylor

**Affiliations:** 1 ICF Consulting Services Ltd, 62 Threadneedle Street, London, EC2R 8HP England; 2 RAND Europe, Eastbrook, Shaftesbury Rd, Cambridge, CB2 8BF England; 3 Breast Cancer Now Ibex House, Fifth Floor, 42–47 Minories, London, EC3N 1DY England; 4 Department of Applied Health Sciences, College of Medicine and Health, University of Birmingham, Birmingham, B15 2TT England; 5 Warwick Medical School, University of Warwick, Coventry, CV4 7AL England

**Keywords:** Women’s health, Primary health care, Reproductive health, Delivery of health care, Integrated

## Abstract

**Background:**

Women’s sexual and reproductive health care in England is fragmented, with multiple providers, access inequalities, and commissioning barriers. In some areas health leaders, often based in primary care settings, have established ‘Women’s Health Hubs’ to improve care and outcomes. The 2022 English Women’s Health Strategy subsequently recommended national implementation of these models. This study aimed to explore Women’s Health Hubs established before 2022 to inform national policy and practice: to describe models, explore experiences of implementation, delivery and care, key features and indicators of success.

**Method:**

Mixed-methods evaluation included an online survey of identified UK hub leaders to identify and describe models and interviews with English regional and national stakeholders (*n* = 13). In-depth work in four purposively selected hubs included interviews with staff working in or connected to the hub (*n* = 40), women using hubs (*n* = 32), focus groups with underserved women in the local community (*n* = 48), and analysis of documents shared by hubs.

**Results:**

Seventeen UK hubs were identified (13 in England). Hubs were diverse in size, maturity, commissioning and delivery models. Primary care leadership and settings predominated. Common services included long-acting reversible contraception and menopause care. Data availability limited the assessment of impact on health and inequalities, though there were examples of short waiting times, improved access to long-acting reversible contraception, and reduced secondary care referral in individual hubs. Women using hubs reported positive experiences. A need for equality in access and avoiding destabilising existing services was emphasised. Hubs were described as potentially improving primary care staff retention. Challenges included fragmented commissioning, workforce shortages, funding, and poorly-integrated infrastructure, including electronic patient records. Perspectives varied regarding the optimal model and leadership, including the role of primary care.

**Conclusions:**

Women’s Health Hubs have potential to integrate and improve women’s care, though there are ongoing challenges in defining and implementing these models. Heterogeneity in models makes extrapolating conclusions difficult. Further evidence is needed of the impact on inequalities, population health and on the wider health system, including unintended or adverse consequences. Tailoring to local context is important. Sustainable national scale-up across England will require funding and time, and primary care professionals and organisations will be central to success.

**Supplementary Information:**

The online version contains supplementary material available at 10.1186/s12875-025-03037-z.

## Introduction

In the UK NHS women’s reproductive health care involves diverse providers, venues and professionals including primary care, gynaecology, maternity and community sexual and reproductive health services. Primary care is usually the first contact, and a key provider of women’s healthcare [1]. Often women’s reproductive health care is not well-integrated into the healthcare system, with inequalities in access [2]. These disparities are associated with ethnicity, deprivation, age, geography, sexuality and gender identity [3–5]. The variation in quality and availability has in part been attributed to a lack of ownership and accountability for women’s health [2, 6–8]. Additional challenges have included gaps in funding, workforce and training, and system pressures [2, 3, 7, 9, 10]. The 2012 English Health and Social Care Act transferred most sexual health service commissioning including contraception into local authorities, while other women’s health services remained within the NHS [2, 3]. This increased service fragmentation in England compared to other UK nations [3]. For example, as a result of the changes many services were only funded to provide intrauterine systems for contraception *or*gynaecological reasons, not both [2, 3]. In 2019 the Royal College of Obstetricians and Gynaecologists (RCOG) recommended comprehensive one-stop community women’s health clinics to improve access to healthcare for women, but this has proved challenging [3].

In response to fragmented care and deteriorating access, clinical leaders in some parts of the UK established integrated women’s health care models, increasingly referred to as Women’s Health Hubs (WHHs) [11]. The UK Primary Care Women’s Health Forum (PCWHF) has produced a toolkit, events and advocacy to support the expansion of WHHs [11].

National scale-up of WHHs was announced in England’s first Women’s Health Strategy in 2022 [12]. The Strategy and the consultation which informed it identified many challenges, including: barriers and inequalities in access to care across the life course; more convenient and joined-up services are needed; women did not feel listened to, particularly for gynaecological symptoms such as pain; there was a need for more diversity in the women involved in service improvement and better care for underserved groups; improved leadership was needed. In 2023 funding was provided to facilitate the establishment of a WHH in each of the 42 Integrated Care Systems (ICS) by Integrated Care Boards (ICBs) in England, led by new Women’s Health Champions appointed in each Integrated Care Board [13].

At the time of publication of the Women’s Health Strategy, there was no agreed definition for WHH models, and little was known about the number, location, characteristics and activities of existing models. However, this is crucial to inform further policy and service developments, particularly in the context of increasing pressures on healthcare services. This paper reports the main findings and additional analysis of a national study [14] which aimed to explore Women’s Health Hub models that were in place prior to the Women’s Health Strategy, describing models, exploring the experiences of implementing, delivering and using WHHs, and defining important features and indicators of success.

## Methods

### Aim

This study aimed to explore Women’s Health Hubs established before 2022 to inform national policy and practice: to describe models, explore experiences of implementation, delivery and care, key features and indicators of success.

### Design

A mixed-methods evaluation combined qualitative and quantitative data. The evaluation was supported by a Stakeholder Advisory Group (comprising ten policy, commissioning and clinical leaders from organisations across the NHS in England) and Women’s Advisory Group (a group of seven diverse women from across England, with a range of lived experience of women’s health issues and NHS care). The evaluation questions are included in supplementary file 3.

### Setting, participants and sampling, data collection

The approach for each component of the study is described separately for 1) national survey, 2) stakeholder interviews, 3) in-depth work in four WHHs. Data collection occurred between April 2022 and March 2023, before national funding was announced to support WHH development. To underpin the work, as WHH were not clearly defined models, we collaboratively developed a ‘working definition’ with our Women’s and Stakeholder groups. WHHs included in the study were required to meet the following criteria: i) based in the community and work at the interface between primary and secondary care and/or the voluntary sector; ii) offer more than a single service (and include the provision of both gynaecological services and contraception) or demonstrate plans to do so; iii) have more than one organisation involved in the process of service delivery, including in design, commissioning and/or provision of care, beyond simply referring in.

#### Survey of UK WHH leads

An online survey of UK hub leaders was conducted to identify and describe models. The number of existing hub models was not known, and no comprehensive list was available, and often services did not have an online presence or adopt the label ‘Women’s Health Hub’. To capture as many models as possible, inclusive and broad nomenclature, ‘integrated community women’s health hubs or services’ was used. Hub leaders were identified and invited to participate by sharing study information via clinical and policy stakeholders including the study Stakeholder Group, known hub leads, professional bodies, through snowballing and via social media. The survey was informed by evaluation questions (Supplementary File 3), scoping work, and was piloted with a consultant in sexual and reproductive health. Survey questions are in Supplementary File 1. A SmartSurvey [15] survey link was sent to all hub leaders identified, with up to three reminders. Data was collected between May to December 2022 to enable identification of as many models as possible. Participants provided consent at the start of the online survey. Responses were excluded if they did not describe a WHH model that met our working definition.

#### Regional and national stakeholder interviews

National policy and practice leaders (*n* = 6) were purposively sampled on the basis of their role in women’s health policy and practice. Regional leaders (*n*= 6 of 7 regions) were sampled purposively and iteratively, to provide a balance of participants in different roles, regions, organisations, and contexts, including individuals working in areas with no WHHs. Participants were identified via clinical and policy stakeholders, including our study Stakeholder Group, and were sent information and an invitation to take part by researchers. Participants included leaders in commissioning, sexual health, primary care, Medical Royal Colleges, the UK Faculty of Sexual and Reproductive Healthcare and PCWHF. Ten scoping interviews with national stakeholders undertaken in January 2022 before the study commenced were also included in analysis, with the approach described elsewhere [11]. Scoping interview questions were similar to the subsequent evaluation, with the addition of questions to inform the design of the main evaluation. Written or verbal consent was recorded prior to data collection.

#### In-depth work in four WHHs

Four of the English hubs identified in the national survey were purposively selected for in-depth analysis. To select sites, the research team collaboratively reviewed the survey data and local population health statistics was used to construct a long-list of WHH site characteristics. A list of over ten dimensions of variation was constructed and presented to Stakeholder and Women’s Groups to agree priority criteria for selection (stage of development of hub, location/geography, clinical leadership, commissioning arrangements, and type of hub model). The research team then met to agree final sites for in-depth work, focusing on the identified priority characteristics while also working to balance other long-list hub characteristics. Data collected at each site included:

- Interviews: Staff working in or connected to the hub (*n* = 9–11 per hub), purposively sampled for maximum variation, identified through discussions with hub leaders and snowballing. WHH patients (*n* = 7–9 per hub), with convenience sampling via clinical gatekeepers who distributed study information to women attending the hub, and predominantly adopted a virtual consent-to-contact approach. In one site clinicians introduced patients interested in taking part to a researcher face-to-face. Written or verbal consent was recorded prior to data collection.
- Focus groups of 5–17 women (one in each hub location) explored perspectives of individuals in populations where WHH leaders/stakeholders reported barriers to healthcare access. One existing community women’s group working with underserved groups was identified in each site, via local healthcare stakeholders, and invited to host a focus group with their service users. This included: a group for women from African backgrounds (*n* = 10); an older women’s exercise group in a underserved community (*n* = 16); a women’s health group serving a rural community (*n* = 5); and a women’s group in an ethnically diverse community (*n* = 17). While we did not sample on the basis of previous hub attendance, when asked during focus groups none of the participants had visited a hub service. Written or verbal consent was recorded prior to data collection.
- Service documents including specifications and performance reports were collected for inclusion in analysis. Hub leaders and local stakeholders including interview participants were asked to identify and share relevant documents with researchers.

Interview and focus group participant characteristics are described in Table 1. Topic guides (see Supplementary File 2) explored subjects including definitions, hub aims and contexts, experiences and achievements. Interviews were face-to-face, online or by telephone (most were online). Two focus groups were conducted face-to-face and two were online. Patient and public participants received a £10 voucher. Interviews and three focus groups were audio-recorded and transcribed verbatim. Contemporaneous notes were taken in one focus group due to women’s preferences.

**Table 1:** In-depth evaluation site participant characteristics

Site	Staff directly involved in hub services e.g. GPs, consultants, administrators, nurses	Other stakeholders e.g. referring GPs, hospital consultant	Women service users	Focus Group participants	Total
1	6	4	8	10	28
2	8	3	7	16	34
3	8	1	8	5	22
4	7	3	9	17	36
Total	29	11	32	48	120

Adapted from Daniel K, Bousfield J, Hocking L, Jackson L, Taylor B. Women's Health Hubs: A rapid mixed-methods evaluation. NIHR Journals Library, 2024 [14]

### Data analysis and triangulation

Descriptive summary statistics were computed for survey data, with content analysis of free text. Qualitative data from all interviews, focus groups and documents were analysed using a team-based qualitative rapid analysis approach to deliver timely findings with methodological rigour [16], drawing on wider methodological literature [17–19]. An initial summary template matrix was informed by the evaluation questions and review of four exemplar transcripts, and was refined iteratively. Data was allocated to separate researchers who reviewed each source (transcripts and documents) and summarised relevant data in a matrix. Matrices were combined, and researchers were allocated different evaluation questions for analysis. Each reviewed relevant charted data and quantitative survey findings, identifying and exploring similarities and differences between sources, participants and contexts, and constructed analytical summaries. Summaries were reviewed, refined and combined by the research team. Participant validation was not undertaken. Researchers met weekly to share and discuss the approach to analysis, insights and emerging findings.

### Reflexivity

The research team are all women, with lived experience of England’s NHS care for women’s health issues. Two are experienced maternity and women’s health researchers, and all have an interest in women’s health. One is a public health clinician and has worked in genitourinary medicine services. Acknowledging their positionality and how it might influence the research, the team employed strategies to embed reflexivity including regular team meetings, actively challenging assumptions and interpretation, and Women’s and Stakeholder Groups who guided the approach and interpretation of findings.

## Results

Integrated quantitative and qualitative findings are presented thematically as follows: participant characteristics; definitions of WHHs; characteristics of WHH models; WHH achievements; the role of primary care; women’s experiences; the role of WHHs in training; WHH implementation.

### Participant characteristics

Seventeen survey responses described WHH models which fit the definition developed with our study Stakeholder and Women’s Groups. Due to the broad nomenclature used in the survey to maximise recruitment (“integrated community women’s health hubs or services”) nine responses did not meet the WHH definition and were therefore excluded, due to not providing or planning to provide contraception services alongside gynaecological care. A further thirteen respondents did not have a WHH model in place and therefore were excluded. The number of UK WHHs was unknown as the study was the first attempt to identify them all, therefore a response rate could not be determined.

Six national policy and clinical leaders and six regional leaders were interviewed. Across the four in-depth sites, 72 participants were interviewed (40 staff, 32 service users) (Table 1), and 48 individuals participated in four focus groups. A detailed description of participant characteristics is reported elsewhere [14]. In the in-depth hub sites, the two thirds of service user participants shared demographic data, and in women’s focus groups one third provided this information. Where data was available, it indicated that both interviews and focus groups with women included individuals with experience of long-term health conditions and different levels of formal education. Interviews included age groups between 18 and 70 years; focus group participants were between 30 and 79 years. Interviews included different sexual and gender identities and diverse ethnic groups. Due to the missing demographic data, the diversity of women participating is likely to be wider than is represented, for example focus groups specifically involved women in community groups for minoritised women.

### WHH definitions

Many WHHs were set up before the term ‘Women’s Health Hub’ was established, they were diverse and no agreed definition or ‘typical’ model was identified. Participants described them as providing holistic women’s health care in the community, beyond standard primary care, while less specialised than secondary care. A ‘hub’ was not necessarily a physical building, but a service offer integrated with local pathways, services and organisations. Provision of more than one service including gynaecology and contraception was a defining feature. There was no consensus on clinical scope. Some participants described joint or integrated commissioning arrangements as a key component. A few suggested WHHs should provide a ‘one-stop-shop’ where women could have all needs addressed. As the term ‘Women’s Health Hub’ was relatively new, it was suggested that some services may already meet the definition of a hub without being labelled as such. While a flexible definition was deemed necessary due to varying contexts, there were concerns about potential confusion.

*“– what is a women’s health hub? What does it absolutely precisely look like? I don’t think we’ve actually defined that yet. I think we bandy around that kind of term and it means as many terms do, different things to different people.”* [Regional interview, commissioner]

A minority of participants discussed the boundaries of ‘women’s health’, and whether the focus of WHHs on women’s reproductive organs was limiting, and that women were *‘… more than just their reproductive organs, but that is a good start.’* [Hub interview, service user] While it was not prominent in the data, this issue was also highlighted by our Women’s Group, who questioned if and how other important services such as mental health care and maternity services would be incorporated into hub models and pathways.

### Characteristics of WHH models

**Fig. 1:**
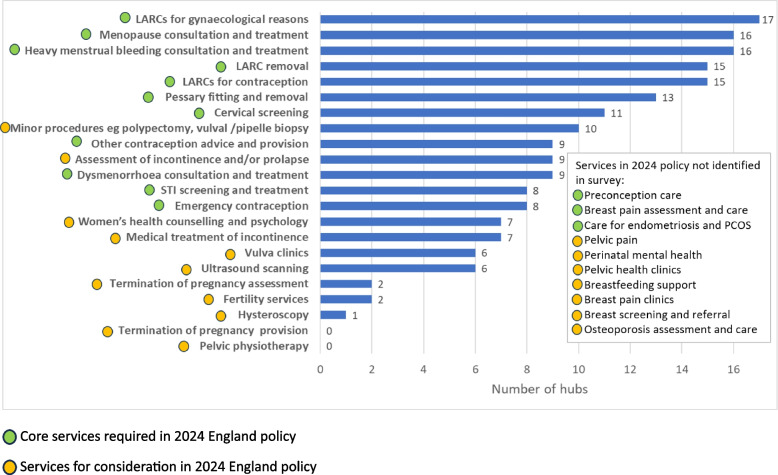
Services offered by UK women’s health hubs identified in national survey compared with 2024 national Core Specification [20]. Adapted from Daniel K, Bousfield J, Hocking L, Jackson L, Taylor B. Women's Health Hubs: A rapid mixed-methods evaluation. NIHR Journals Library, 2024 [14]

Table 2 summarises features of the 17 hubs identified. Figure 1 presents the services offered. The most common services provided were: long-acting reversible contraception (LARCs, intrauterine systems and implants) for gynaecological (*n* = 17) or contraceptive (*n* = 15) purposes or removal (*n* = 15); consultation and treatment for menopause (*n* = 16) and heavy menstrual bleeding (*n* = 16). Most hubs had plans to expand their offer (*n* = 13). None offered pelvic physiotherapy or termination of pregnancy. Fertility services (*n* = 2) and hysteroscopy were rarely offered (*n* = 2). Figure 1 is annotated to indicate the aspects of the national Women’s Health Hubs Core Specification which was published after the study was completed and which all hubs were working towards. No WHHs in our survey were providing the full range of core services required in this policy, and three core services were not reported as being offered by any hub: preconception care, breast pain assessment and care, and care for endometriosis and polycystic ovary syndrome (PCOS). However, it is likely that PCOS and endometriosis care may have been provided in many hubs: the survey did not explicitly name these conditions as options.

**Table 2:** Characteristics of UK Women’s Health Hubs identified in national survey

**WHH characteristic**	Description	Frequency
UK nation	England Northern Ireland Scotland Wales	13 4 0 0
Venues***	GP practices Hospital Sexual health clinic Community NHS trust Pharmacy-based clinic	13 3 2 1 1
Clinical background of hub leader	GP Sexual and Reproductive Health Consultant Consultant Gynaecologist (secondary care)	12 4 1
Commissioning model	Multiple commissioners involved: Clinical Commissioning Group (CCG)* and local authority** Commissioned by CCG or equivalent only Commissioned by local authority only No formal commissioning arrangements NHS hospital trust commissioned No response	7 5 1 2 1 1
Delivery models***	One-stop shop (several services available under one roof) Hub and spoke (provide complex services in a central ‘hub’, linked to a network of community ‘spokes’) Virtual e.g. online consultations, education sessions Other No response	9 6 3 2 1
Clinical and support roles working in the hub	GP with a special interest in women’s health Administrators Healthcare assistants Hospital gynaecology consultants, practice nurses GPs Community sexual and reproductive health (SRH) consultants Specialist nurses Community SRH trainees GP trainees Counsellors Community SRH specialists Advanced nurse practitioners Nursing assistants Data analysts Community gynaecologist Integrated sexual health speciality doctor	13 11 7 5 3 3 2 2 2 1 1 1 1 1 1 1

Adapted from Daniel K, Bousfield J, Hocking L, Jackson L, Taylor B. Women's Health Hubs: A rapid mixed-methods evaluation. NIHR Journals Library, 2024 [14]

^*^The survey referred to CCGs which were the statutory NHS commissioning bodies in England responsible for planning and commissioning health services, when identified hubs were established

^**^Organisation responsible for public services in an area

^***^Respondents could select more than one option

Most hubs were in England (*n* = 13) with some in Northern Ireland (4) and none in Scotland or Wales. Organisational footprints varied, e.g. single or multiple Primary Care Networks (PCNs, groups of GP practices serving 30,000 to 50,000 people). Eleven served populations larger than 100,000, and no hub in England served an entire ICS population. Findings suggested that the majority of areas of the UK were not served by a WHH.

WHH aims were diverse, with no clear patterns identified among different models and contexts. All survey respondents who answered this question (*n* = 14) cited aims of improving choice, reducing waiting times, minimising appointments needed to address problems, and reducing secondary care referrals. Most hubs operated from multiple venues (*n* = 16), usually primary care or community clinics. Venues included dedicated hub spaces, satellite clinics (e.g. based in GP surgeries), and virtual activities (e.g. telephone triage or online consultations). Other common features included launch within the past five years (*n* = 9) and GP clinical leadership (*n* = 12). Commissioning approaches varied, including multiple organisations (*n* = 7), single organisations (*n* = 7), and no formal arrangements (*n* = 2).

While many described models as one-stop shops, it was rare for multiple services to be offered during the same appointment, and where this occurred it was opportunistic rather than a core service. LARC was offered for both gynaecological and contraceptive reasons (*n* = 15), or gynaecological indications (*n* = 2). All had GP referral pathways, and five offered some self-referral. Eleven included triage in the pathway for referrals (this was unclear in 3, not described in 3). Some offered weekend (*n* = 5) or evening (*n* = 5) services. All offered face-to-face clinics, with two also offering online/video appointments and one virtual group consultations.

The most common group working in hubs was GPs with a special interest in women’s health (*n* = 13), though there was no consistent workforce approach. In nine hubs, the workforce included GPs alongside gynaecology and/or sexual and reproductive health consultants, while eight were GP-only models. While there was variation between models, there was consensus that services should be developed according to local needs and context: no optimum model was identified.

*“It depends on their population needs…With a very old population maybe you want to start concentrating on pessary care, a younger population you might be wanting to sort of look at early menopause care. I think people have got to tailor make it to their population …” *[Scoping interview, GP].

### Achievements and outcomes

Hubs had provided access and care for thousands of women. Heterogeneity in data prevented assessment and comparison of activity, outcomes and impacts. Outcomes were usually reported at individual hub level. The impact on wider system activity and outcomes was not possible to assess. Participants highlighted a need for standardised data collection, reporting and electronic patient records integration. Limited available outcome data suggested low onward referral rates (range 5–14%, three hubs). Triage was usually within days and appointments within a few weeks (four hubs). In one area, referrals to secondary care gynaecology by non-hub GPs reduced by 14%. One site evidenced an 8.5% increase in LARC fitting in its local area following WHH implementation.

### The role of primary care in WHHs

WHHs may change the role of primary care in women’s health care, with potential implications including primary care leadership in women’s health, destabilisation of services, training needs and workforce. In this evaluation of early models, most hubs were GP-led, with services delivered in GP practices, often by GPs. Interviewees suggested that new hubs could be developed incrementally from existing primary care services.

*“..so our vision is very much to expand into delivering other services. So those at the top of the list would be – we already deliver some cytology, some sexual health screening, but those things that we really feel well positioned to continue to deliver in a bigger scale, and directly through the hubs, would be things like pessary fittings, menopause care. Possibly things like cervical polyp removals*.” [Hub interview, leader, GP].

While multidisciplinary involvement was universally welcomed, perspectives varied regarding leadership and there was no consensus. Reported advantages of GP leadership included holistic care underpinned by generalist life course expertise with understanding and connection to primary care. Consultants were described as able to manage more diverse and complex clinical issues, with better access to additional expertise, resources and training, fewer competing priorities, and more women’s health clinical governance and management expertise. Consultant SRH roles included explicit system-networking and population health expertise and responsibilities. Individuals from a primary care background or connected to GP-led models tended to favour GP leadership, while those with gynaecology or sexual and reproductive health backgrounds often favoured consultant leadership.

*“I think that an SRH consultant, based on their training, is the right person to lead on it, not deliver it. We need GPs, pharmacists, nurses, nursing assistants, physicians’ associates, everybody involved in delivering it. But I think an SRH consultant lead is really valuable…Because the last thing you want is for patients to be constantly referred onto someone else. So there will be some GPs who can do a lot of the medical gynaecology, but very few GPs who can do the whole spectrum you know, like managing vulva etcetera.” *[Hub interview, leader, Sexual and Reproductive Health Consultant].

It was proposed that WHHs could be GP-led, with consultants providing expert clinical support and oversight. Some suggested that identifying an enthusiastic local women’s health leader was key, and that clinical backgrounds could vary. While leadership discussions focused on doctors, one participant highlighted the potential for nursing and allied health professional roles.

*“…[a WHH] might be led by a nurse to start with or even a pharmacist or a paramedic or a GP, but then… it should grow from there to get a bigger team.”* [National interview, GP].

Some concerns were raised regarding potential over-centralisation and destabilisation of primary care. Some suggested that primary care can meet many needs without referral, and there were fears that GPs could be de-skilled, or that funding and work could be diverted to WHHs.

*“…everybody’s very keen but when…push comes to shove, general practice may feel that this may deskill them if all of this happens in a women’s health hub”* [National interview, Consultant Gynaecologist].

A few participants reflected that primary care-based hubs could facilitate access, being familiar and conveniently located in communities. It was suggested that digital hub access e.g. through online self-referral, could exclude some women.

General practice workforce concerns were prominent, with a finite pool of health professionals to deliver primary care and women’s health services, and challenges in retaining staff. It was suggested that clinicians may leave primary care to work in hubs, though others indicated that combining routine primary care practice with WHH sessions may improve retention.

*“[Health Service Leader] thinks, rightly I think, you’ll keep a workforce if you can do some days – that’s why I’m still in general practice, you know I do some days at the GP coalface which I enjoy-ish, and then I have fun doing my women’s health stuff.”* [*National* interview, GP].

Potential explanations for WHH roles encouraging retention of clinical staff included being able to provide longer appointments, ability to focus on an area of interest and personal passion (women’s health), access to training and support, and a sense of achievement. Overall staff working in hubs reported positive experiences, pride and fulfilment in interviews, and in local staff satisfaction surveys in exemplar sites.

### Women’s experiences

Service users reported predominantly positive experiences of access and care, feeling listened to, having more time than in other settings, and receiving more personalised care, with a woman-centred focus and expertise.

*“It felt personal. I didn’t feel like I was just being rushed through, as you do in the doctor’s now. I felt that that that person was taking proper time to sit down with me, ask me lots of questions and allowed me to ask lots of questions back…”* [Hub interview, service user].

There were some suggestions for improvement, including information about follow up.

*“She’s not my GP…I’m not sure if I am one of her patients so I’m not really sure where I stand with that…And whether I should be going to my GP again…So I’m a little bit confused as to who’s leading me with this.”* [Hub interview, service user].

Few hubs reported involving women in hub design, but participants emphasised the importance of creating WHHs informed by what women want and need. Addressing inequalities was a frequent objective of WHH leaders. Some women in focus groups were concerned about potential access inequalities, citing current variation in access to GP appointments.

*“It takes you a month, yeah, it can take you weeks even now to access a GP. No appointments at GPs, nothing. It’s been two months now. I’m crying to get a GP and this is something really, really worrying because by the time you go and the GP tells you ‘oh, it’s too late’, imagine*?” [Women’s Focus Group].

While women using hub services reported positive experiences, they were rarely aware that the service was a Women’s Health Hub. Most women in interviews and focus groups did not recognise the term ‘women’s health hub’ though the concept was welcomed once explained.

*“I think women need to know that these services are there because from this little meeting, it looks like a lot of women are not even aware that the service is there*.” [Women’s Focus Group].

Views of existing WHH models could not be explored in focus groups as women did not know about them, but participants discussed the challenges they faced in accessing care, and what a WHH should look like. Women and staff suggested approaches to encourage uptake, including: awareness-raising activities; convenient, accessible or familiar locations including mobile clinics; flexible appointments; childcare; language interpreting.

*“The hub has to be near the people so they can reach it with transport. And also access for those women who have disabilities. So there has to be more accessibility…the location of it is also important, it has to be local. Also the transport is accessible.” *[Women’s Focus Group].

### The role of WHHs in training

Some WHHs offered training to local staff, had mapped local skills, and had implemented plans to improve provision. Shortages of trained staff presented challenges in some areas. Some roles required accredited training (e.g. LARC fitting). Perspectives varied regarding the qualifications required to work in a hub, including that it could be a barrier to competent staff joining WHH teams.

*“I am very nervous that people will expect training and qualifications that A, won’t be appropriate, B will take a long time and C just will be too costly and people won’t do them.”* [National interview, GP].

Nursing and allied health professions were involved in many sites. In some sites the NHS Additional Roles for Reimbursement Scheme enabled this at no extra cost. Task-shifting was being trialled in some areas (e.g. pharmacists and physicians’ associates trained to fit LARCs), though some suggested a need for evaluation of its feasibility and safety. Some WHHs delivered activities to upskill local GPs, including: training in GP practices; clinical observation; case-based support and discussions; feedback on referrals. Reported benefits of training and support included reductions in inappropriate hub referrals, gynaecology referrals, and increased management of problems in routine primary care.

*“…that’s now come in as part of the model that GPs can phone [name] one day a week for advice …”* [Hub interview, leader, Consultant in Sexual and Reproductive Health].

### Practicalities in WHH implementation

Leadership was repeatedly highlighted as important. Sites had clinical and non-clinical leaders committed to improving local women’s health, who worked tirelessly and collaboratively across boundaries to make the case for, set up and deliver WHHs. It was suggested that expansion of WHH models would be more difficult in areas without champions with similar skills, passion and capacity.

*“So, the service in [place] it works because [name] has been absolutely dedicated and gone above and beyond what normally healthcare professionals and GPs do in terms of setting up and keeping the gynae service going.”* [Hub interview, GP working in hub].

Reported barriers included limited funding and resources (e.g. equipment, physical space); varying stakeholder engagement; system pressures; competing priorities; and infrastructure, particularly poorly-integrated clinical records systems. Sharing of records between WHHs and other services varied widely depending on the WHH provider and organisational boundaries, though no model explored was able to seamlessly view and share records across all parts of the system, which impacted on communication between services, ordering tests, prescribing, and monitoring of service performance. Workarounds were described to ensure effective communication, such as having two different electronic patient record systems open during a consultation and manually transferring information. A small number of clinicians expressed concerns that open access self-referral WHH models may create demand which could not be met. Leaders described substantial efforts to overcome barriers, often with practical support or resource from partners (e.g. PCN or pharmaceutical companies).

*“…there were so many different obstacles to unravel … one of the major obstacles was IT, but the PCN model enabled that cross referral…so they solved a lot of the IT issues.”* [Hub interview, local Consultant Gynaecologist].

Funding and commissioning barriers were common. Fragmented commissioning presented challenges, particularly providing integrated LARC services for both contraception and gynaecological reasons, and moving activity from secondary care (often funded by block contracts) to community services. Workarounds were often described, including providing care without seeking reimbursement, though this was perceived as unsustainable and a threat to WHH expansion.

*"Secondary care isn’t always prepared to release the funding…ring pessaries for gynae, we've been doing it without getting payment for many, many years …I know they're struggling to get funds released for menopause as well… [the hospital] can't meet their demand, their waiting list is huge for the menopause clinic, last time I tried to refer somebody there was an eighteen month wait.”* [Hub interview, GP].

The creation of ICSs and ICBs in 2022 offered potential to expedite hub scale-up across systems. However, women’s health competed for attention with other priorities such as emergency care pressures, and at the time of data collection ICBs had no designated women’s health leads.

*“..there are other things that you find that are being prioritised over and above women’s health. What would be really good is that if we could really, really prioritise this, but almost have that kind of, you know, national approach and national drive to take things in a certain direction”* [Regional interview, Commissioner].

Concern was also raised by leaders regarding the sustainability of WHHs, and long-term funding and support. This was echoed in one of the Focus Groups where women described a community contraception clinic closing.

*“Maybe it could reach up to 2 or 3 years and then close down, you know? So [what I would want] it’s to leave it, to continue this service to grow, not to be closed down.”* [Women’s Focus Group].

*“It must be about 10 years ago they shut them [community contraception clinics] down because then you had to start going to the doctor’s, but then you’re taking an appointment off someone to get your pill.”* [Women’s Focus Group].

## Discussion

### Summary

This evaluation identified 17 WHHs, predominantly in England. They were diverse in size, maturity, and commissioning and delivery models, though there were some services common across models, particularly LARCs. Impacts were challenging to evaluate and compare with the available data. Women who used hubs had good experiences, though a need to ensure all women could access hubs was emphasised. Key challenges included continued fragmented commissioning, workforce shortages, funding, and poorly-integrated infrastructure. While GP leadership and primary care settings predominated, perspectives varied regarding the optimal approach. WHHs aimed to integrate and improve pathways, and may improve retention of primary care staff, however, there were fears about destabilisation of existing provision.

### Strengths and limitations

This evaluation combined breadth and depth in data collection, triangulating different sources. It had strong stakeholder engagement, including a Women’s Advisory Group and Stakeholder Advisory Group. Some WHHs may not have been included: models were challenging to locate with no agreed definition or register of known services. The use of a rapid analysis approach enabled timely reporting of findings about these emerging models to policymakers, though some granular detail may have been missed. Data availability meant that there were sometimes difficulties in obtaining a detailed understanding of hub processes and outcomes, and evaluation of impact was beyond scope. Findings predominantly reflected stakeholder perspectives in areas where enthusiastic leaders established models and so may provide a more positive assessment of WHHs.

### Comparison to existing literature

WHHs are one of many examples of integrated care models in the literature [21–26]. Our findings align with evidence that models work best when they are flexible and locally tailored [19, 20, 27–29]. Others have also noted the challenge of balancing flexibility with a standardised ‘core’ offer across different models and populations [30]. The WHHs in our evaluation were developed ‘bottom up’ by local leaders, though more recently the Department of Health and Social Care in England has since defined core services for English WHHs [17]. While existing WHH models focused on reproductive health, research with primary care professionals emphasised a need for a more holistic definition of women’s health where “*Women are not just their reproductive organs and their hormonal cycles and the challenges in navigating the role, specialisation and service boundaries for GPs and others in women’s health provision*.” [1]. The predominantly GP referral models in most WHHs did not mitigate NHS GP access barriers and inequalities [1].

Measuring outcomes and impact is notoriously difficult in integrated care models where data is not accessible across organisations [31]. The limited evidence that WHHs may reduce waiting times, referrals and improve patient experience aligns with wider evidence [28]. Challenges observed in WHH implementation are consistent with the literature [32]. The pace of implementation and impact of new care models can be challenging to align with rapid policy timescales [28]. WHHs were able to provide the expertise and time that is often not available in primary care [1]. However, changing established care pathways may have unintended consequences, potentially destabilising other services [33, 34].

### Implications for research and practice

England’s Women’s Health Strategy evidences policymakers’ commitment to women’s health [12]. WHHs are relatively new integrated care models with evolving definitions, scope, clinical leadership and boundaries, which may substantially impact on primary care’s role in women’s reproductive health in the English NHS. While the early hub models identified in this study included many services stipulated in the 2024 national Women’s Health Hubs Core Specification [17], none offered the full range of services which Integrated Care Boards in England have been asked to implement by December 2024, and some were not offered at all such as breast assessment and care, therefore further evidence is required to understand how the more comprehensive policy specification is implemented in practice. Other services such as termination of pregnancy were not offered in the included hubs, though assessment was offered in rare cases. Women are likely to perceive abortion care as the domain of an integrated women’s health service, though currently it is delivered by specialist providers. This is another example of fragmentation of services in England which is crucial to consider in integrating services and pathways going forward to ensure timely, equitable access, particularly as demand has increased substantially [35]. The hubs identified also focused on reproductive health. The need to offer a comprehensive, life course service for women’s health is increasingly recognised [36]. Our study Women’s Group emphasised the need to consider how hubs would integrate other services such as maternity, mental health or cardiovascular services.

Most clinical leaders will be familiar with ‘what works’ to support WHH implementation: strong inclusive leadership, needs assessment, stakeholder engagement including patients, and tailoring models to local needs and context, and enablers such as IT infrastructure. Consideration of unintended consequences on existing services and patients will be important. Women may need appropriate information and support to access WHHs equitably. While English ICBs were allocated funding to establish WHHs, sustainable, scalable commissioning solutions are not yet clear. This is likely to require transfer of funding from other services, and joined-up commissioning approaches, in the context of competing priorities and substantial financial and workforce pressures in primary care and the wider NHS in England.

Since the completion of this evaluation, specific ringfenced WHH implementation funding and targets have been discontinued, and a new 10-year Health Plan for England has been published [37, 38]. The 10-year Plan includes a focus on moving more care into primary care settings, and which cites WHHs as an exemplar of local neighbourhood health services. The UK government has announced its intention to turning the commitments in the women’s health strategy into tangible actions, though as identified in a report by the Royal College of Obstetricians and Gynaecologists, detail is needed on how the 10-year Plan will be aligned with the Women’s Health Strategy, including WHHs as part of the shift to neighbourhood health services.

## Conclusions

This study provides a detailed analysis of the first examples of Women’s Health Hubs in England that were developed by enthusiastic leaders prior to the 2022 Women’s Health Strategy. Implementation may be more challenging in new locations. Our evaluation of early models highlights hubs’ potential, but the impact on population-level care, outcomes, services and costs requires further exploration. Ongoing national scale-up offers the opportunity to evaluate expansion of these models to understand their role in improving and integrating care. Primary care professionals and organisations are central to the successful implementation and delivery of WHHs in the communities they serve, as lynchpins of an integrated, sustainable life course approach to women’s reproductive health.

## Supplementary Information

Supplementary Material 1.

Supplementary Material 2.

Supplementary Material 3.

## Data Availability

The datasets used and/or analysed during the current study are not available due to ethics restrictions.

## Abbreviations

NHS: National Health Service
RCOG: Royal College of Obstetricians and Gynaecologists
PCWHF: Primary Care Women’s Health Forum
WHH: Women’s Heath Hub
ICS: Integrated Care System
ICB: Integrated Care Board
GP: General Practitioner
PCOS: Polycystic Ovary Syndrome
CCG: Clinical Commissioning Group
SRH: Sexual and Reproductive Heath
LARC: Long-acting reversible contraception
