# Supplementary material for: Community Women's Health Hub models in England: a mixed methods evaluation
Source: BMC Prim Care. 2025 Dec 16;26:398. doi: 10.1186/s12875-025-03037-z (PMC12709742; doi:10.1186/s12875-025-03037-z)
Supplement: Supplementary file 1 — Supplementary Material 1. [file 12875_2025_3037_MOESM1_ESM.docx]

**Women's Health Hub Evaluation Survey Questions**

1. **Participant information**

***1. Has your local area put in place a community women’s health service, sometimes known as a Women’s Health Hub (even if it is in development/not fully implemented)? ****

|  | Yes |
| --- | --- |
|  | No |
|  |  |

***2. Which nation are you primarily based in? ****

|  | England |
| --- | --- |
|  | Scotland |
|  | Wales |
|  | Northern Ireland |

***3. Please state your job title/s ****

|  |
| --- |

***4. Please state your job role in relation to your local Women’s Health Hub (or equivalent service) ****

|  |
| --- |

***5. Please provide your email address, so that we can contact you to clarify any details in your responses, and to share the final report if you wish to receive it. ****

|  |
| --- |

***6. Which local authority area does your Women’s Health Hub (or equivalent service) serve? ****

|  |
| --- |

1. **Background**

***7. What is the current status of your local Women’s Health Hub/service? Please select one of the following***

|  | In development (yet to launch) |
| --- | --- |
|  | The hub is currently operational, with plans to expand further |
|  | The hub is currently operational with no plans for further expansion at this time |
|  | The hub is no longer operational (i.e. previous hub has now closed/paused) |
|  |  |

***8. When (year) was your local Women’s Health Hub launched, or when is launch planned to occur?***

|  |
| --- |

***9. If the hub launch occurred in stages, please describe***

|  |
| --- |

***10. What is the name of your local Women’s Health Hub (note, it may not be called a ‘hub’)?***

|  |
| --- |
|  |

***11. Which organisations and populations are served by your local Women’s Health Hub? (e.g. what is the name of the local authority area covered)?***

| Primary Care Network name/s | \|  \| \| --- \| |
| --- | --- | --- |
| Clinical Commissioning Group (or equivalent) name/s | \|  \| \| --- \| |
| Local Authority name/s | \|  \| \| --- \| |
| Integrated Care System name/s | \|  \| \| --- \| |

***12. Which organisations and populations are served by your local Women’s Health Hub? (e.g. what is the name of the local authority area covered)?***

| Primary Care Cluster name/s | \|  \| \| --- \| |
| --- | --- | --- |
| Local Health Board name/s | \|  \| \| --- \| |
| Local Authority name/s | \|  \| \| --- \| |

***13. Which organisations and populations are served by your local Women’s Health Hub? (e.g. what is the name of the local authority area covered)?***

| GP Cluster name/s | \|  \| \| --- \| |
| --- | --- | --- |
| Integration authority name/s | \|  \| \| --- \| |
| Local Authority name/s | \|  \| \| --- \| |
| Health Board name/s | \|  \| \| --- \| |
|  |  |

***14. Which organisations and populations are served by your local Women’s Health Hub? (e.g. what is the name of the local authority area covered)?***

| GP Federation name/s | \|  \| \| --- \| |
| --- | --- | --- |
| Local Authority name/s | \|  \| \| --- \| |
| Integrated Care Partnership name/s | \|  \| \| --- \| |
| Local Commissioning Group name/s | \|  \| \| --- \| |

***15. What patient population does your local Women’s Health Hub serve?***

|  | All women in the area |
| --- | --- |
|  | Specific groups of women only (please specify below) |

Comments:

|  |
| --- |

***18. What is the estimated total population covered by your local Women’s Health Hub (note total population, not only women)***

|  | <10,000 |
| --- | --- |
|  | 10,000-29,999 |
|  | 30,000-49,999 |
|  | 50,000-99,999 |
|  | 100,000+ |
|  | Don't know |
|  | Other (please specify):   \|  \| \| --- \| |

Comments:

|  |
| --- |

***16. How many of the following organisations/bodies are involved in planning, commissioning and delivery of your local Women’s Health Hub/service? Please provide numbers or state where not known***

| GP practices referring women to the Health Hub | \|  \| \| --- \| |
| --- | --- | --- |
| GP practices providing services to the Hub | \|  \| \| --- \| |
| Primary Care Network/s (PCNs) | \|  \| \| --- \| |
| Clinical Commissioning Group/s (or equivalent) | \|  \| \| --- \| |
| Local Authority/ies | \|  \| \| --- \| |
| Integrated Care System/s (ICSs) | \|  \| \| --- \| |
| NHS acute trust/s | \|  \| \| --- \| |
| NHS community trust/s | \|  \| \| --- \| |
| Private sector provider/s | \|  \| \| --- \| |
| Voluntary sector organisations | \|  \| \| --- \| |
| Women’s groups | \|  \| \| --- \| |

***17. Why was your local Women’s Health Hub established?***

|  |
| --- |

***18. What are the current or future objectives of your local Women’s Health Hub (please tick all that apply)?***

|  | Current objective | Future aspiration | Not current objective/ future aspiration | Don’t know |
| --- | --- | --- | --- | --- |
| Improve women’s health outcomes (in general) |  |  |  |  |
| Provide holistic care to women |  |  |  |  |
| Improve women’s experience of accessing care |  |  |  |  |
| Improve choice for women |  |  |  |  |
| Provide care closer to home |  |  |  |  |
| Reduce waiting times |  |  |  |  |
| Reduce the number of appointments women require for a problem |  |  |  |  |
| Enable multiple issues to be addressed in the same appointment |  |  |  |  |
| Achieve financial efficiencies |  |  |  |  |
| Reduce unplanned pregnancy |  |  |  |  |
| Increase uptake of Long-acting Reversible Contraceptives (LARCs) |  |  |  |  |
| Reduce secondary care use/make secondary care more efficient |  |  |  |  |
| Address current gaps in local GPs’ women’s health provision |  |  |  |  |
| Reduce inequalities in access and care for women |  |  |  |  |
| Integrate services/reduce fragmentation |  |  |  |  |
| Improve/increase focus on prevention in women’s health |  |  |  |  |
| Educate/upskill local GPs in women’s health |  |  |  |  |
| Educate and empower women to self manage and seek help as needed |  |  |  |  |
| Provide new/additional women’s health services |  |  |  |  |
| Other (please state below) |  |  |  |  |

Comments:

|  |
| --- |

***19. Which of the following best describes the key person/role driving the establishment of your local Women’s Health Hub? Please select one of the following, or if the hub involved equal collaboration between local leaders, please tick all that apply***

|  | Local GP/s with interest in this area |
| --- | --- |
|  | Sexual and Reproductive Health Consultant(s) |
|  | Secondary care Gynaecologist(s) |
|  | Local authority commissioner(s) |
|  | Clinical Commissioning Group/s (or equivalent) |
|  | Don’t know |
|  | Other (please specify):   \|  \| \| --- \| |

**C. Commissioning and funding**

***20. Which of the following best describes the commissioning arrangements for your local women’s health hub?***

|  | There are no formal commissioning arrangements in place |
| --- | --- |
|  | Co-commissioned between local authority and CCG (or equivalent) |
|  | Commissioned by local authority only |
|  | Commissioned by CCG (or equivalent) only |
|  | Don’t know |
|  | Other (please specify):   \|  \| \| --- \| |

***21. What sort of contract is in place (if any)? Please tick all that apply***

|  | Locally Enhanced Service |
| --- | --- |
|  | Direct contract with primary care |
|  | Contract via secondary care provider |
|  | Block contract with pooled budget |
|  | Other (please specify):   \|  \| \| --- \| |

***22. Has your local Women’s Health Hub received any additional funding from any source?***

|  | Yes |
| --- | --- |
|  | No |

***23. Who provided the additional funding?***

|  |
| --- |

***24. How much additional funding has been allocated?***

|  |
| --- |

***25. Is the current funding arrangement permanent or short-term?***

|  | Permanent |
| --- | --- |
|  | Short-term |
|  | Don't know |

Comments:

|  |
| --- |

***26. Is any of the funding COVID-related?***

|  |
| --- |

***27. Please describe the clinical leadership and governance structure for your hub, including the key roles and key accountability structures***

|  |
| --- |

***28. Which of the following best describes your model of clinical leadership? Please select one***

|  | GP-led |
| --- | --- |
|  | Sexual and Reproductive Health Consultant-led |
|  | Gynaecology Consultant-led |
|  | Shared leadership model (please state between who below) |
|  | Don’t know |
|  | Other (please specify):   \|  \| \| --- \| |

Comments:

|  |
| --- |

**D. The service and pathways**

***29. How would you describe your local Women’s Health Hub model? Please select all that apply***

|  | Hub and spoke |
| --- | --- |
|  | One stop shop |
|  | Pop up |
|  | Virtual |
|  | Other (please specify):   \|  \| \| --- \| |

***30. What services are currently provided by your local Women’s Health Hub? Please select all that apply***

|  | Long-acting Reversible Contraceptives (LARCs) for contraception |
| --- | --- |
|  | Long-acting Reversible Contraceptives (LARCs) for gynaecological reasons |
|  | Long-acting Reversible Contraceptive (LARC) removal |
|  | Emergency contraception |
|  | Other contraception advice and provision (not specific to LARCs or emergency contraception) |
|  | Cervical screening |
|  | Heavy menstrual bleeding consultation and treatment |
|  | Dysmenorrhoea consultation and treatment |
|  | Menopause consultation and treatment |
|  | Sexually transmitted infection screening and treatment |
|  | Women’s health counselling and psychology |
|  | Ultrasound scanning |
|  | Hysteroscopy |
|  | Fertility services |
|  | Assessment of incontinence and/or prolapse |
|  | Medical treatment of incontinence |
|  | Pessary fitting and removal |
|  | Pelvic physiotherapy |
|  | Termination of pregnancy assessment |
|  | Termination of pregnancy provision |
|  | Vulva clinics |
|  | Minor procedures eg polypectomy, vulval /pipelle biopsy |
|  | Other (please specify):   \|  \| \| --- \| |

Comments:

|  |
| --- |

***31. What information do you provide to women in your area about the services provided by the hub?***

|  |
| --- |

***32. Please describe how women are referred to your local Women’s Health Hub service***

|  |
| --- |

***33. Are Women’s Health Hub referrals managed through an electronic system?***

|  | Yes, entirely |
| --- | --- |
|  | Yes, partially |
|  | No |
|  | Don’t know |

Comments:

|  |
| --- |

***34. Does your Women’s Health Hub service triage referrals received?***

|  | Yes |
| --- | --- |
|  | No |
|  | Don't know |

***35. Who undertakes triage, and how does the process work?***

|  |
| --- |

***36. Where are patient records held for care delivered within the Women’s Health Hub?***

|  | Within GP records (accessible to those delivering Hub services) |
| --- | --- |
|  | In a separate system (e.g. secondary care) which can link with GP record and other IT systems as needed (please state system below) |
|  | In a separate system (e.g. secondary care) which does not link to GP record (please state system below) |
|  | Don’t know |
|  | Other (please specify):   \|  \| \| --- \| |

Comments:

|  |
| --- |

***37. Can staff order tests electronically in your Hub using IT systems?***

|  | Yes |
| --- | --- |
|  | No |
|  | Some tests can be ordered electronically, but not all |
|  | Don't know |

***38. How are tests ordered non-electronically and which tests does this relate to?***

|  |
| --- |
|  |

***39. Can staff prescribe electronically in your hub using IT systems?***

|  | Yes |
| --- | --- |
|  | No |
|  | Don't know |

***40. How are prescriptions placed?***

|  |
| --- |

***41. Does your local Women’s Health Hub have a dedicated website?***

|  | Yes |
| --- | --- |
|  | No |
|  | Don't know |
|  |  |

***42. Please share the link of the website***

|  |
| --- |

***43. Are women able to access the Women’s Health Hub website:***

|  | Yes | No | Don't know |
| --- | --- | --- | --- |
| For information about the services offered by the Hub |  |  |  |
| For information about women’s health more generally |  |  |  |
| For self-referral to the Women’s Health Hub |  |  |  |

Comments:

|  |
| --- |
|  |

***44. Please list the venues providing Women’s Health Hub services in your area:***

|  | Venue (including digital/online/remote services) | Service provided | Professionals working in hub at this site | How often services provided (e.g. 2x 4 hour sessions per week) |
| --- | --- | --- | --- | --- |

Comments:

|  |
| --- |

***45. Does your local Women’s Health Hub provide services:***

|  | Yes | No | Don't know |
| --- | --- | --- | --- |
| In evenings |  |  |  |
| At the weekend |  |  |  |

Comments:

|  |
| --- |

***46. Is it possible for women to see more than one healthcare professional during the same visit to a Women’s Health Hub service?***

|  | Yes |
| --- | --- |
|  | No |
|  | Don't know |

Comments:

|  |
| --- |

***47. What sort of appointments does your hub offer? Please select all that apply***

|  | Face to face appointments |
| --- | --- |
|  | Telephone appointments |
|  | Online appointments |
|  | Group video consultations |
|  | Other (please specify):   \|  \| \| --- \| |

**E. Workforce**

***48. Which of the following professionals work in your local Women’s Health Hub? Please select all that apply. Please provide approximate numbers and whole time equivalent for each (where possible)***

|  | Number of individuals | Whole time equivalent |
| --- | --- | --- |
| Hospital gynaecology consultants | \|  \| \| --- \| | \|  \| \| --- \| |
| Hospital gynaecology associate specialists | \|  \| \| --- \| | \|  \| \| --- \| |
| Hospital gynaecology trainees | \|  \| \| --- \| | \|  \| \| --- \| |
| Community sexual and reproductive health consultants | \|  \| \| --- \| | \|  \| \| --- \| |
| Community sexual and reproductive associate specialists | \|  \| \| --- \| | \|  \| \| --- \| |
| Community sexual and reproductive health trainees | \|  \| \| --- \| | \|  \| \| --- \| |
| GPs | \|  \| \| --- \| | \|  \| \| --- \| |
| GPs with special interest in women’s health | \|  \| \| --- \| | \|  \| \| --- \| |
| GP trainees | \|  \| \| --- \| | \|  \| \| --- \| |
| Genitourinary medicine (GUM) consultants | \|  \| \| --- \| | \|  \| \| --- \| |
| Genitourinary medicine (GUM) associate specialists | \|  \| \| --- \| | \|  \| \| --- \| |
| Genitourinary medicine (GUM) trainees | \|  \| \| --- \| | \|  \| \| --- \| |
| Practice nurses | \|  \| \| --- \| | \|  \| \| --- \| |
| Specialist nurses | \|  \| \| --- \| | \|  \| \| --- \| |
| Advanced Nurse Practitioners | \|  \| \| --- \| | \|  \| \| --- \| |
| Physiotherapists | \|  \| \| --- \| | \|  \| \| --- \| |
| Nursing Assistants | \|  \| \| --- \| | \|  \| \| --- \| |
| Healthcare Assistants | \|  \| \| --- \| | \|  \| \| --- \| |
| Physician Assistants/Associates | \|  \| \| --- \| | \|  \| \| --- \| |
| Care assistants | \|  \| \| --- \| | \|  \| \| --- \| |
| Pharmacists | \|  \| \| --- \| | \|  \| \| --- \| |
| Ultrasonographers | \|  \| \| --- \| | \|  \| \| --- \| |
| Counsellors | \|  \| \| --- \| | \|  \| \| --- \| |
| Administrators (with dedicated time for the hub work) | \|  \| \| --- \| | \|  \| \| --- \| |
| Data analysts (with dedicated time for the hub work) | \|  \| \| --- \| | \|  \| \| --- \| |
| Other (please state below) | \|  \| \| --- \| | \|  \| \| --- \| |

Comments:

|  |
| --- |

***49. Do you have community sexual and reproductive health consultants in your local area?***

|  | Yes |
| --- | --- |
|  | No |
|  | Don't know |

Comments:

|  |
| --- |

***50. Do you have an integrated Sexual Health Service in your area?***

|  | Yes |
| --- | --- |
|  | No |
|  | Don't know |

Comments:

|  |
| --- |

***51. Does your hub provide training to healthcare professionals?***

|  | Yes |
| --- | --- |
|  | No |
|  | Don't know |

***52. Please describe what type of training is offered and for which roles***

|  |
| --- |

***53. How do you determine competence of staff delivering service (if at all)? For example, do you have a bespoke training/competency framework?***

|  |
| --- |

***54. Do your hub GPs undertake any clinical work in the hospital, e.g. as part of a reciprocal arrangement where hospital staff also work in the hub?***

|  | Yes |
| --- | --- |
|  | No |
|  | Don't know |

Comments:

|  |
| --- |

**F. Performance management/monitoring**

***55. Which (if any) of the following data are currently used to monitor your Women’s Health Hub activity/quality? Please select all that apply.***

|  | Currently in place | Planned in future |
| --- | --- | --- |
| Number of clinics delivered |  |  |
| Number of women attending |  |  |
| Waiting times between referral and appointment |  |  |
| Long-acting Reversible Contraceptives (LARC) fitting rates |  |  |
| Termination of pregnancy rates |  |  |
| Number of referrals to secondary care |  |  |
| Patient experience feedback |  |  |
| Hub staff experience/feedback |  |  |
| Local non-hub staff experience/feedback |  |  |
| GP training/upskilling numbers |  |  |
| Budget/spend |  |  |
| Other (please state below) |  |  |

Comments:

|  |
| --- |

**G. Additional information**

***56. How does your hub address inequalities and widen reach?***

|  |
| --- |

***57. Have you undertaken any public involvement work in the development or delivery of your hub?***

|  | Yes |
| --- | --- |
|  | No |
|  | Don't know |

***58. Please describe how you have undertaken any public involvement work in the development or delivery of your hub***

|  |
| --- |

**H. Facilitators and barriers**

***59. What are the top three key factors which facilitated implementation of your hub***

| 1 | \|  \| \| --- \| |
| --- | --- | --- |
| 2 | \|  \| \| --- \| |
| 3 | \|  \| \| --- \| |

***60. What are the top three challenges which impacted on implementation of your hub?***

| 1 | \|  \| \| --- \| |
| --- | --- | --- |
| 2 | \|  \| \| --- \| |
| 3 | \|  \| \| --- \| |

**I. Documents**

***61. Are you able to share any of the following with the evaluation team?***

|  | Business case |
| --- | --- |
|  | Service specification |
|  | Commissioning plans/Locally Enhanced Service details etc |
|  | Needs assessment/local intelligence work that informed hub development (e.g. engagement work with women and staff, analysis of routine data) |
|  | Local evaluations/case studies |
|  | Performance dashboard |
|  | Patient pathway diagram |
|  | Organogram or other diagram illustrating how the relationships between organisations work |
|  | Governance, management and/or leadership information |

***62. Is there any further information you would like to share regarding your local hub?***

|  |
| --- |

**J. Future contact**

***63. Would you be willing to be contacted to clarify any of the responses in this survey?***

|  | Yes |
| --- | --- |
|  | No |

***64. Are you willing to be contacted about further evaluation work involving Women’s Health Hubs (agreeing does not commit you to taking part in future work)?***

|  |  | Yes |
| --- | --- | --- |
|  |  | No |

***65. Please tick this box if you would like to receive a copy of the final report in 2023***

|  | Yes, I would like to receive a copy of the final report |
| --- | --- |
