# Supplementary material for: Community Women's Health Hub models in England: a mixed methods evaluation
Source: BMC Prim Care. 2025 Dec 16;26:398. doi: 10.1186/s12875-025-03037-z (PMC12709742; doi:10.1186/s12875-025-03037-z)
Supplement: Supplementary file 2 — Supplementary Material 2. [file 12875_2025_3037_MOESM2_ESM.docx]

**Evaluation of Women’s Health Hubs**

**National interviews topic guide**

1. Please describe your (national-level) role in relation to women’s, sexual or reproductive health?
2. What do you understand a Women’s Health Hub to be?/How would you define a Women’s Health Hub? What sort of services are not included in your definition of a hub (where do hubs begin, and other services end)?

Prompts: what types of integration do you consider possible within a WHH

1. How did the idea of Women’s Health Hubs come about? What are they intended to address?

Prompts: health inequalities, postcode lottery, fragmentation of care, poor service access, lack of choice, Covid backlog, service duplication.

1. How do these Women’s Health Hubs fit with wider policy and other national/regional/place-based developments such as PCNs and ICSs?

Prompts: links with other strategies e.g. sexual and reproductive health action plan, fit with ICSs/plans, links with other regional structures and more localised PCN development, priority in landscape, visibility, links with wider strategic health and social care plans, joining up across region e.g. leadership, ways of working.

1. What sorts of models exist for women’s health hubs, and how do they compare to other hub models (e.g. family hubs, perinatal mental health hubs, community diagnostic hubs)?

Prompts: leadership, commissioning, hub/spoke/one-stop-shop etc., different stakeholder perspectives. Should WHH models be standardised across different areas/services?

1. What are your thoughts on funding / commissioning models for Women’s Heath Hubs and on how challenges around funding / commissioning arrangements can be addressed? Probe for examples of where solutions have been found, and their views on the additional support / arrangements that might be needed.
2. What are your reflections about the workforce and training needs to deliver hubs?

Prompts: formal/informal training, which staff lead/deliver services, how this impacts on wider services (e.g. if GPs working in hubs), different stakeholder perspectives.

1. What are Women’s Health Hubs intended to achieve, and do different stakeholders prioritise different things? What are your reflections on whether Women’s Health Hubs are making progress towards this/any evidence of early impacts?

Prompts (intentions): improve women’s health outcomes, provide holistic care, improve access for women, improve choice, provide care closer to home, reduce waiting times, reduce inequalities in access and care, reduce secondary care use, address current gaps in local provision, integrate services/reduce fragmentation, improve women’s health prevention, educate and empower women to self-manage and seek help, educate/upskill local healthcare professionals, reduce the number of appointments required for a problem, provide new/additional services

Prompts (early impacts): impact on their service/work/experiences of delivering care to women, impact on local health system, changes to partnership working/relationships between services, changes in access to services, changes in demand for services [inc around system], changes in numbers attending appointments, changes in waiting times, freeing up time in specialist clinics, changes in awareness of women’s health issues, patient satisfaction.

1. What do you think are the key outcomes/markers of success for hubs, and how could/are these (be) measured?

Prompts: number of clinics delivered, number of women attending, waiting times, number of LARC fittings, termination of pregnancy rates, patient experience feedback, hub staff experience feedback, GP training/upskilling numbers, budget/spend.

1. How do you think Women’s Health Hubs are intended to address health inequalities?

Prompts: improve choice, improve access, focus on local issues/gaps in provision, target underserved populations, provide holistic care, provide more equitable care across an area.

1. How does local context impact on the development of Women’s Health Hubs? Or, what are the main causes of (or points of) variation in the way in which women’s health services are developed and provided?

Prompts: leadership, staffing, funding, geography, commissioning arrangements, service history

1. What are your reflections on hub leadership approaches?

Prompts: different types of leadership – impact of primary care vs SRH or gynae led, prevalence, benefits and drawbacks of each, any particular models that are perceived to be more/less important, different stakeholder perspectives.

1. How do/could women’s health hubs integrate with and impact on the wider health and care system in their local area? Do women’s health hubs integrate with other type of hub models across the NHS and beyond (e.g. family hubs, perinatal mental health hubs, community diagnostic hubs)?
2. What are your reflections on how women’s health hubs can be scaled up, spread and/or sustained? What work is happening currently to facilitate this? What could hinder progress with this?
3. What do you consider to be the future key areas of focus / considerations for future working for WHHs?

Prompts: service offer, commissioning, funding, service integration, policy alignment, spread and scale, evidencing impact.

1. As you know, recently, the new women’s health strategy for England was published. What are your reflections on the strategy, and in particular the focus on the development of women's health hubs? How is the strategy impacting on implementation of hubs?

Prompts: drive/plans for hubs locally, funding, support, expansion of current models, impact (or future impact) on future work around women’s health

1. What are your thoughts about how the focus on women’s health hubs in the strategy will drive and support local work to develop hubs across the UK?
2. Are you aware of any national/government guidance and support for women’s health hubs? (e.g. funding, guidelines for commissioning). What national/government support do you think may be needed?
3. Lastly, are there any topics you would like to raise that I haven’t asked you about?

**Regional interviews topic guide**

1. Please describe your regional-level role in relation to women’s, sexual or reproductive health (as participants may have multiple roles)?
2. Could you please briefly tell us a little about your wider regional context?

Prompts: ICSs, strategic partnerships, place based working, regional leadership and investment, future plans.

1. Could you please tell us about community-based women’s health service provision in your region? (Clarify that this includes any and all services providing women’s health services, which could include primary care, specialist sexual and reproductive health services, gynaecology, physio, pharmacists etc.)

Prompts: availability, structure, commissioning, workforce, investment/finances, services offered, population covered.

1. What do you think works well in relation to the provision of community-based women’s health services in your region?

Prompts: availability and location of services, choice, workforce, training, waiting times, investment/finances, commissioning.

1. What are the main challenges in relation to the provision of community-based women’s health services in your region?

Prompts: availability and location of services, workforce, training, waiting times, investment/finances, commissioning, fragmentation, inefficiencies in system service delivery, gaps in provision, inequalities in access and care.

1. Have you heard of Women’s Health Hubs before we contacted you about this interview? (If not briefly define as necessary)
2. Are you aware of any Women’s Health Hubs or similar service models in your region? If so, could you please tell us a little about them.

Prompts: high-level objectives/drivers behind it, how many, where are they, population served, type of model e.g. GP led, services covered, any early outcomes/impacts. Are they called Women’s Health Hubs?

1. How do these Women’s Health Hubs fit with other regional/place-based developments such as PCNs and ICSs?

Prompts: fit with ICSs/plans, links with other regional structures and more localised PCN development, priority in landscape, visibility, links with wider strategic health and social care plans, joining up across region e.g. leadership, ways of working.

1. (If there aren’t any Women’s Health Hubs in their region), do you know why Women’s Health Hubs have not been established in your region so far?
2. We know that local context can play a big role in establishing new models of care and their success. Which contextual features in your area do you think are most likely to be influential in the development and establishment of Women’s Health Hubs?
3. Are you aware of any regional/local evaluations of Women’s Health Hubs?

Prompts: If yes – do you know who is leading the evaluation? Do you know who has funded this?

1. Lastly, are there any topics you would like to raise that I haven’t asked you about?

**Staff interviews topic guide**

Please adapt the term used for ‘hubs’ according to the local context/insights from leads, local teams may use other terms such as ‘enhanced service’

**Hub staff**

Background

1. Can you briefly describe your professional background and current role/s?
2. Please describe your role in relation to the local Women’s Health Hub/enhanced service?

Prompts: length of time in role, roles/responsibilities in relation to the hub, how much of their time is spent working on/in the hub.

1. What do you understand a Women’s Health Hub to be?

Prompts: what is it? how does it differ from other services? Links with local strategies/commissioning plans

Set up and implementation – ask if relevant (some staff may not be involved in this)

1. Could you please describe the local model(s) [set up] for your hub?

Prompts: Services offered, scope, how is it commissioned, population covered (size and specifics), type of model (e.g. GP led), structure e.g. one stop shop, hub and spoke, clinical leadership, number of staff, team composition and staff mix, staff skills, employing organisations, governance structure, how was this model decided upon? Who was involved in deciding the local model? If so, how? Why was this particular approach chosen? Can the hub be accessed by all women or is there criteria for use? If so, what happens to women who fall outside of this criteria? Are particular groups of women prioritised?

1. What is the leadership model for the hub? How does this fit with local commissioning arrangements and governance structures?
2. How has the Women’s Health Hub been funded/resourced?

Prompts: What funding was provided? Was this enough? Is this continuous or one-off funding? Who provided the funding? Is this new funding or reallocated monies from elsewhere? How have resources been allocated? Is any funding Covid-19 related? Any alternative resources, in-kind costs. How have challenges in commissioning and funding Womens’ Health Hubs been resolved in your area? How will the Hub be funded going forwards? Are there any cost implications for other services? (e.g. less or more activity for other services due to the presence of the Hub? Are there any monitoring processes in terms of costs/ resource use? Are you limited in the activities you can undertake due to funding constraints?

1. Why was the Women’s Health Hub set up?

Prompts: Improve women’s health outcomes, provide holistic care, improve access for women, improve choice, provide care closer to home, reduce waiting times, reduce inequalities in access and care, reduce secondary care use, address current gaps in local provision, integrate services/reduce fragmentation, improve women’s health prevention, educate and empower women to self-manage and seek help, educate/upskill local healthcare professionals, reduce the number of appointments required for a problem, provide new/additional services.

Was funding/ costs a consideration in setting up the hub? Please explain?

1. How is the hub intended to meet local needs and address health inequalities?

Prompts: improve choice, improve access, focus on local issues/gaps in provision, target underserved populations, provide holistic care, provide more equitable care across an area.

1. Which sectors/services have been involved in hub set up and delivery?

Prompts: GP practices, CCGs, PCNs, local authorities, ICSs, Trusts, voluntary sector organisations, private sector providers, women’s groups/public involvement.

1. [if not already described] Can you describe the role of the hub in local workforce training and development?
2. Which staff are offered training, how does this work, what is being achieved currently, any future plans.

Experiences

1. What are your reflections on how hub set up and implementation went/is going?

Prompts: what have been the main barriers and enablers to the implementation of the hub?

1. [hub delivery staff only] What have been your experiences of delivering services as part of the hub?

Prompts: who they work with, integration with other services, sharing of data and information, referrals, triage processes, advertisement/communication about hub services, types of appointment offered, use of IT systems/patient records, how does it differ from delivering services outside of the hub, how does the service address the needs of different women, satisfaction with working as part of the hub. How does the hub link with other/wider services? Barriers and enablers to hub delivery.

1. Please describe a ‘typical’ patient journey following referral to your hub?

Prompts: referral speed and source, communication with patient, waiting times, staff seen at hub and services provided, whether more than one professional/problem can be seen in one appointment, where women go for care, communication/integration with other health services, follow up.

1. [staff involved in commissioning only] What have been your experiences of the commissioning process for the hub?

Prompts: What are the local commissioning arrangements? Has there been integration or co-commissioning across services? What contracts are in place? How does it differ from commissioning other services for women’s health/commissioning services prior to introduction of the hub? Have relationships been established as a result of commissioning hub services? **What helped/hindered in establishing local commissioning arrangements?** What are the commissioning arrangements likely to be in the future? Are there any issues associated with commissioning due to the integration of services that are traditionally commissioned separately e.g. sexual health vs. gynaecological services?

1. How do you think the hub has been received by women?

Prompts: Awareness and views on hub, quality of service, any patient feedback/reflections, levels of engagement, do women know it is a hub or is it just a more streamlined version of care? are there any groups of women (in intended population) who you feel aren’t yet accessing/engaging with the hub? Why might that be?

1. Looking back on everything so far, what has gone well?

Prompts: different ways of delivering care to women, access, communication, commissioning arrangements, staffing, joint working between different services, training, referrals and criteria, funding/resource availability, digital working, links with wider strategies/plans. Examples of good practice. What helped these aspects to go well/ why do you think they went well?

1. Looking back on everything so far, what has gone less well?

Prompts: different ways of delivering care to women, access, communication, commissioning arrangements, staffing, joint working between different services, training, referrals and criteria, funding/resource availability, digital working, links with wider strategies/plans. What prevented these aspects from going well/ why do you think they didn’t go well?

Performance and outcomes

1. What are referral and uptake patterns for your hub services?

How many women are referred/attending, does this vary across the patch? Is it changing (i.e. improving) over time?

Are you aware of any enablers and barriers to referrals and to uptake?

1. What impact is the hub having? If so, what? How has the presence of the hub impacted on other local services?

Prompts: Changes to partnership working/relationships between services, changes in access to services, changes in demand for services [inc around system], changes in numbers attending appointments, changes in waiting times or waiting lists, freeing up time in specialist clinics, changes in awareness of women’s health issues, patient satisfaction. Could impact be improved? If so, how?

1. How is the hub meeting local needs and addressing health inequalities?

Prompts: Targeting particular groups of women, focusing on local issues, improving access, linking with wider services, involving local women. If not, why do you think it is not?

1. How are you measuring outcomes for the services being delivered as part of the hub?

Prompts: Which datasets/systems are you using? Which measures/indicators are being used? [e.g. number of clinics, women attending, waiting times, number of LARC fittings, patient experience feedback] Who is responsible for this? How frequently is this information collected? How is this information analysed and used? E.g. to inform decisions. Which measures are most useful/important? Are there any outcome measures required as part of commissioning requirements?

1. Do you think the hub will achieve its intended outcomes? Can you explain why?

Future plans and sustainability

1. How do you see the work of the hub developing in the future?
2. Do you think the hub will be sustained? If yes, how? If not, why not?

Finally

1. What advice would you give to other areas developing a Women’s Heath Hub?
2. Lastly, are there any topics you would like to raise that I haven’t asked you about?

**Wider staff**

Background

1. Can you briefly describe your professional background and current role/s?
2. Please describe your role in relation to the local Women’s Health Hub/enhanced service?

Prompts: length of time in role, roles/responsibilities in relation to the hub

1. What do you understand a Women’s Health Hub to be?

Prompts: what is it? how does it differ from other services? Links with local strategies/commissioning plans

1. [senior strategic stakeholders] How do these Women’s Health Hubs fit with place-based developments in your area, such as PCNs and ICSs?

Prompts: fit with ICSs/plans, links with other structures and more localised PCN development, priority in landscape, visibility, links with wider strategic health and social care plans, joining up across region e.g. leadership, ways of working.

1. [senior strategic stakeholders] How do Women’s Health Hubs fit with the plans and direction of travel detailed in the new women’s health strategy [if published]?

Prompts: fit with plans, development of new infrastructure/services, visibility, influence of strategy on local plans, funding, joining up across region e.g. leadership, ways of working.

1. [E.g. for GPs not involved in hub delivery] Can you describe the patient pathway for your local Women’s Health Hub?
2. What have been your experiences of working with the hub?

Prompts: integration with other services, sharing of data and information, triage processes, advertisement/communication about hub services, staff engagement, differences to pre-hub introduction. Have there been any barriers or enablers to efficient/positive working with the hub?

1. Are you seeing any evidence of early impacts from the hub? If so, what?

Prompts: Impact on their service/work/experiences of delivering care to women, impact on local health system, changes to partnership working/relationships between services, changes in access to services, changes in demand for services [inc around system], changes in numbers attending appointments, changes in waiting times, freeing up time in specialist clinics, changes in awareness of women’s health issues, patient satisfaction. Could impact be improved? If so, how?

1. How is the hub meeting local needs and addressing health inequalities?

Prompts: If so, how? If not, why do you think it is not?

Future plans and sustainability

1. How would you like to see the work of the hub developing in the future?
2. Lastly, are there any topics you would like to raise that I haven’t asked you about?

**Service user interviews topic guide**

Please adapt the term used for ‘hubs’ according to the local context/insights from leads, local teams may use other terms such as ‘enhanced service’. Please also adapt as needed as women may not recognise having received care as part of a ‘hub’ or may have only received support for one issue and so their care does not look/feel different.

Background

1. Please tell us a little about yourself

Prompts: employment, age, interests

1. In general, what do you think women’s health services should encompass/offer?

Prompts: different services to include (and split of gynae and contraception), coverage of mental and physical health, models of delivery, staffing, anything else needed

1. Please could you share with us what you know about this service?

Prompts: What is this service? Who is it run by? How have you come to have an appointment here today?

1. Did you know that this service was called a hub?

Prompts: If no, what do you know this service as? If yes, when did you first become aware of this hub? E.g. at the time of referral/or beforehand, if a self-referral how did they become aware?

1. [If not already covered] What services are you aware of that are offered by the hub?

Prompts: different services available e.g. long-acting reversible contraception, smear tests, menopause management

1. [If not already covered] How were you put in contact with this local hub/service?

Prompts: referral process, who provided the information/self-located, how was an appointment booked, ease of booking an appointment, waiting times for an initial appointment

1. [If not already covered] Is this your first appointment here/at the hub?

Prompts: number of previous appointments, services accessed

1. [If not already covered] If you are comfortable to share this information, which services have you received at the hub?

Communication and information

1. [If not already covered] What communication, if any, and in what format, did you have with the hub before your appointment?

Prompts: what did they know prior to the appointment, format of information provided, quality of information provided, website availability, ease of locating information

1. How would you like to be contacted by the hub in future? What form of communication is easiest for you?

Prompts: telephone, text, email, letter, any alternatives

1. Have you been informed if you will receive any updates on treatment plans or any next steps? If so, in what format will this be? E.g. verbal update, letter to patient, letter to GP
2. How does the hub/service provide wider education or information on women’s health issues?

Prompts: leaflets, advertising for wider services, availability of staff to speak to, courses

Experiences

1. More generally, what are your thoughts on what was good about the appointments/care you have had at the hub/service?

Prompts: interactions with staff, waiting times, communication before and during appointment, ease of access/convenience, childcare provision, appointment times, wait times, satisfaction with care, resolution of symptoms/problems, follow up support, how joined up were appointments/times (if a woman received multiple services).

1. What has been less good about the appointments/care you have had at the hub/service?

Prompts: interactions with staff, waiting times, communication before and during appointment, ease of access/convenience, childcare provision, appointment times, wait times, satisfaction with care, resolution of symptoms/problems, follow up support, how joined up were appointments/times (if a woman received multiple services).

1. What could be done to improve the appointments/care you have had at the hub/service?

Prompts: interactions with staff, waiting times, communication before and during appointment, ease of access/convenience, childcare provision, appointment times, wait times, satisfaction with care, resolution of symptoms/problems, follow up support, how joined up were appointments/times (if a woman received multiple services).

1. Have you attended appointments for support with women’s health issues elsewhere? How does your care today compare with other services you have used, and if so, how?

Prompts: efficiency, number of appointments required for a problem, staff seen, waiting times, quality of interactions, efficiency, level of care (holistic?).

1. How satisfied are you with the care you have received at [insert name of hub/service]?

Prompts: quality of care, interactions with staff, sense of being listened to, what has been good or bad about the care received, feelings of being appropriately supported.

1. How has the hub/service linked with other services that you have used?

Prompts: links with GP, secondary care, specialists, sexual health services, other local services. How has this worked?

1. Were there any things that made it difficult for you to attend the hub? Did anything help you to attend?

Prompts: child care provision, travel times/journey/accessibility/parking arrangements, provision of public transport, flexibility of and timing of appointments, stigma, cultural factors, support from healthcare professionals, cost.

1. How would you access this service again in the future if you need to? (Clarify that they may not know this)

Outcomes

1. What difference has the hub/service made to the care you have received for women’s health issues?

Prompts: Changes in access to services (and locations), changes in waiting times, changes in awareness of women’s health issues, patient satisfaction (how happy women are with the service), changes in number of appointments received. Were there any things that helped, or made it more difficult, for you get the care you need/ find a solution to your health problem? (e.g., feeling heard, the specialists/healthcare professionals you spoke with, the format of your appointment – individual, group, educational, diagnostic, virtual, face-to-face).

1. [For those who have attended a hub before/ had an appointment a while ago], what happened after you attended the hub?

Prompts: Did you receive any follow up support or care? Was this needed? Did you receive any information on treatment plans/next steps e.g. verbal, written letter to GP or patient? Aftercare, mental health support. Were you referred to any other services or signposted elsewhere?

1. How do you think that the hub/service is able to respond to different women’s health needs that someone may have?

Prompts: range of services, availability of appointments, appropriateness of care, staffing levels, ; anything missing; what's not joined up but you think should be?

1. How do you think the hub/service meets local needs for women in your community?

Prompts: improving choice, improving access, focus on local issues/gaps in provision, targeting underserved populations, provide holistic care, providing more equitable care across an area. Could the hub improve the way it meets local needs for women in your community? If so, how?

Finally

1. Is there anything else that could be done to improve this hub/service in any way? If so, how?
2. Lastly, are there any topics you would like to raise that I haven’t asked you about?

**Community group interviews (focus group) topic guide**

Please adapt the term used for ‘hubs’ according to the local context/insights from leads, local teams may use other terms such as ‘enhanced service’. Please also adapt as needed as women may not recognise having received care as part of a ‘hub’ or may have only received support for one issue and so their care does not look/feel different.

Background

1. Please tell us a little about yourself

Prompts: name, employment, age, interests, what primarily brings you to this community group?

Current context and service use

1. If you had a women’s health related issue, for example, required a smear test, needed a coil fitting or needed support with the menopause [adapt as appropriate for women present e.g. could also include heavy menstrual bleeding, fertility, contraception advice]– what steps/where would you currently go to access help?
2. If you felt like you needed more information or education on a woman’s health related issues (such as requiring a coil fitting, cervical screening or the menopause), where would you go to access this and why?
3. What do you think about the women’s health services that are currently available in your area/community? [reiterate area of the hub as women's understandings of area could differ]

Prompts: different types of services available, accessibility, location, staffing, use

Women’s health hubs

1. We’re doing some research about Women’s Health Hubs, which bring different services such as those for contraception, menopause management and cervical screening together to work more closely in a local area and provide women’s health services in the community.

There is a hub in your area called [NAME], which offers [INSERT] [INSERT ANY MORE RELEVANT LOCAL DETAILS]. You may have heard it called [INSERT ANY OTHER NAMES] instead. What do you know about it? [make it clear that they may not have heard about it, or know much and that this is ok]

Prompts: If so, how and when did they become aware of the hub/service? Did you know it was called a hub? Have you used it at all?

[If some interviewees share that they have attended the hub, please incorporate some questions from the topic guide for interviews with women service users (focused on experience).]

1. [Describe local service in more detail e.g. how it’s accessed, when, where, provision (if not done for question above)] – what do you think of this kind of service?
2. Is it something you would consider using?

Prompts: If so, why? If not, why not? E.g. considerations around access, childcare provision, travel times/journey/accessibility/parking (costs)/public transport, flexibility of appointments, stigma, cultural factors, perceived support from healthcare professionals

1. What, if anything, would help you to use this service?

Prompts: awareness of service, communication, childcare provision, travel times/journey/accessibility/parking (costs)/public transport, flexibility of appointments, support with relevant cultural factors, support from healthcare professionals.

1. What, if anything, might make it more difficult for you to use a service/hub like this?

Prompts: childcare provision, travel times/journey/accessibility/parking (costs)/public transport, flexibility of appointments, stigma, cultural factors, interactions with healthcare professionals.

Finally

1. More generally, what, if any, improvements would you like to see in relation to your local women’s health services?

Prompts: location, integration of services [explain which e.g. contraception and smear test], ease of access, education provision, flexibility of appointments

1. Lastly, are there any topics you would like to raise that I haven’t asked you about?
