# Supplementary material for: Community Women's Health Hub models in England: a mixed methods evaluation
Source: BMC Prim Care. 2025 Dec 16;26:398. doi: 10.1186/s12875-025-03037-z (PMC12709742; doi:10.1186/s12875-025-03037-z)
Supplement: Supplementary file 3 — Supplementary Material 3. [file 12875_2025_3037_MOESM3_ESM.docx]

**Supplementary file 3**

**Evaluation questions:**

1. What are WHHs, and is there variation in how stakeholders name and define them?
2. How many WHHs have been established or are in development across the UK, where are they, and what are their characteristics, including models of structure, commissioning and delivery?
3. Why have WHHs been implemented, and how are they intended to address health inequalities?
4. What have WHHs achieved to date? How do WHHs achieve this?
5. What are the experiences and perspectives of staff regarding WHH set-up, commissioning, funding, implementation and delivery?
6. What are the experiences and perspectives of women who have used hub services?
7. How are WHHs’ performance, outcomes and costs measured, and how might they be measured in future?
